# Circulating eNAMPT as a biomarker in the critically ill: acute pancreatitis, sepsis, trauma, and acute respiratory distress syndrome

**DOI:** 10.1186/s12871-022-01718-1

**Published:** 2022-06-15

**Authors:** Christian Bime, Nancy G. Casanova, Sara M. Camp, Radu C. Oita, Juliet Ndukum, Vivian Reyes Hernon, Dong Kyu Oh, Yansong Li, Phil J. Greer, David C. Whitcomb, Georgios I. Papachristou, Joe G. N. Garcia

**Affiliations:** 1 Department of Medicine, University of Arizona Health Sciences, Tucson, AZ USA; 2 University of Ulsan College of Medicine, Asan Medical Center, Seoul, Korea; 3 US Army Institute of Surgical Research, San Antonio, TX USA; 4 University of Pittsburgh School of Medicine, Pittsburgh, PA USA; 5 Ariel Precision Medicine, Pittsburgh, PA USA; 6 Ohio State University College of Medicine, Columbus, OH USA

**Keywords:** NAMPT, eNAMPT, ARDS, Pancreatitis, Trauma, Sepsis, Biomarker

## Abstract

**Background:**

Nicotinamide phosphoribosyltransferase (NAMPT) exhibits dual functionality – as an intracellular enzyme regulating nicotinamide adenine dinucleotide metabolism and as an extracellular secreted protein (eNAMPT) to function as a cytokine regulator of innate immunity via binding to Toll-Like receptor 4 and NF-κB activation. In limited preclinical and clinical studies, eNAMPT was implicated in the pathobiology of acute respiratory distress syndrome (ARDS) suggesting that eNAMPT could potentially serve as a diagnostic and prognostic biomarker. We investigated the feasibility of circulating eNAMPT levels to serve as a biomarker in an expanded cohort of patients with ARDS and ARDS-predisposing conditions that included acute pancreatitis, sepsis, and trauma with comparisons to controls.

**Methods:**

A total of 671 patients and 179 healthy controls were included in two independent cohorts. Plasma and serum eNAMPT levels were quantified using one of two complementary Enzyme-linked Immunosorbent Assays. After log base 2 variance stabilizing transformation of plasma/serum eNAMPT measurements, differences between healthy controls and each disease cohort were compared using linear regression or a generalized estimating equation (GEE) model where applicable. Complementary analyses included sensitivity, specificity, positive predictive values, negative predictive values, and the area under the receiver operating curve.

**Results:**

Compared to controls, circulating eNAMPT levels were significantly elevated in subjects with acute pancreatitis, sepsis, trauma, and ARDS (all *p* < 0.01). In the acute pancreatitis cohort, circulating eNAMPT levels positively correlated with disease severity (*p* < 0.01).

**Conclusions:**

Circulating eNAMPT levels are novel biomarker in the critically ill with acute pancreatitis, sepsis, trauma, and/or ARDS with the potential to reflect disease severity.

**Supplementary Information:**

The online version contains supplementary material available at 10.1186/s12871-022-01718-1.

## Introduction

Viral and bacterial sepsis, trauma, and acute pancreatitis are inflammatory disorders that commonly precede the development of acute respiratory distress syndrome (ARDS), a lung disorder characterized by an intense inflammatory response which affects ~ 500,000 patients annually in the United States [1]. ARDS patients rapidly develop acute hypoxemic respiratory failure requiring mechanical ventilation with 30–40% mortality from the resulting multi-organ system failure [2]. Morbidity and mortality from ARDS have dramatically increased worldwide due to the ongoing SARS-CoV-2/COVID-19 pandemic [2]. ARDS diagnosis encompasses a heterogeneous pathobiology, further exacerbated by imprecise clinical and radiographic diagnostic criteria [3]. There is a compelling unmet medical need for biomarkers with pathophysiologic relevance to guide subject stratification for enrollment in future clinical trials investigating personalized ARDS therapies.

Previous genomic–intensive approaches have identified potentially novel therapeutic targets in sepsis and acute inflammatory lung disorders including ARDS [4–10]. Among ARDS is the gene encoding nicotinamide phosphoribosyl transferase (*NAMPT*), a novel candidate gene in ARDS [4]. NAMPT protein exhibits dual functionality – as an intracellular enzyme regulating nicotinamide adenine dinucleotide metabolism and as an extracellular protein (eNAMPT) functioning as a cytokine regulator of innate immunity via binding to Toll-Like receptor 4 (TLR4) [11] and NF-κB activation. eNAMPT was among a panel of six biomarkers shown to be highly predictive of 28-day mortality in ARDS [12] and included Interleukin-6 [IL-6], Interleukin-8 [IL-8], Interleukin-1 receptor antagonist [IL-1RA], macrophage migration inhibitory factor [MIF], and Angiopoietin-2 [Ang-2]. The role of eNAMPT as a diagnostic and prognostic biomarker has previously been studied in critically ill patients with sepsis [12–14]. In one study, eNAMPT levels alone did not distinguish survivors from non-survivors but exhibited good diagnostic accuracy when a member of the six-biomarker panel [13]. Karampela et al. showed that baseline circulating eNAMPT was a better discriminator of sepsis and septic shock compared to C-reactive protein and procalcitonin and may distinguish survivors from non-survivors [14]. In this current study, we investigated whether circulating eNAMPT levels were significantly elevated in patients with ARDS and in ARDS-predisposing conditions such as acute pancreatitis, sepsis, septic shock, and trauma. Furthermore, we sought to determine the optimal cutoff point for plasma eNAMPT levels to determine disease severity in critically ill patients with ARDS -inducing conditions.

## Methods

### Sources of data

**Fig. 1:**
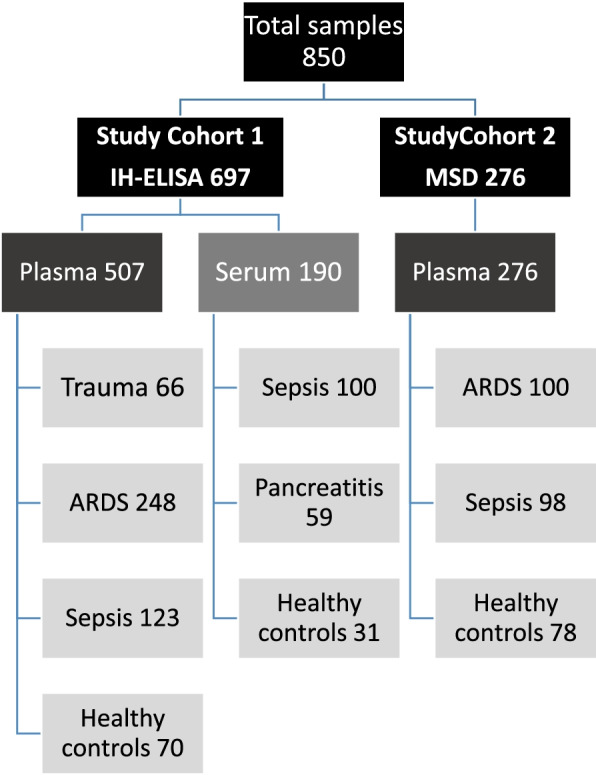
Study Cohorts, specimen allocations, and diagnoses included in the First and Second Study Cohorts

A total of 671 blood specimens from patients with ARDS, sepsis, acute pancreatitis, or trauma and 179 healthy controls were included in this study. All study grouped specimens were independently obtained. Informed consent was obtained from all the participants and protocols were approved by the Institutional Review Boards at each center. The first cohort included a combination of serum and plasma samples, analyzed separately. The serum pools consisted of 59 subjects with acute pancreatitis/ 11 healthy controls from the University of Pittsburgh (IRB#STUDY20060223) [15, 16] and 100 subjects with sepsis/20 controls from Asan Medical Center, Seoul, Korea (Approval #2001–0001). The plasma pools consisted of 66 trauma subjects from the US Army (IRB# L-12–004), 123 subjects with sepsis and septic shock, and 248 ARDS subjects from the NIH Fluid and Catheter Treatment Trial (FACTT) study [17], the University of Arizona (IRB#1312168664R001) and University of Illinois (IRB #20,120,192) along with 70 controls from the University of Arizona (IRB#1312168664R001). A second study cohort included 276 plasma specimens (100 ARDS, 98 sepsis, and 78 controls) from the University of Arizona (IRB #1312168664R001). Figure 1 details the flow diagram of assay type, specimen type, and diagnoses that were included in both cohorts.

### Participants

All patients with ARDS met diagnostic criteria per the Berlin Definition [3]. Patients with sepsis met the Consensus definition for sepsis guidelines [18] at enrollment. Acute pancreatitis was diagnosed based on the International Association of Pancreatology (IAP)/American Pancreatic Association (IAP/APA) guidlines [19]. Grading of the severity of acute pancreatitis was determined based on the Revised Atlanta Classification [20, 21]. Due to the limitations of various repositories included, it was not possible to differentiate between the severity of ARDS, trauma, and sepsis in the same manner performed for acute pancreatitis.

### Blood collection and measurement of eNAMPT

We utilized two complementary assays to measure eNAMPT in samples from multiple well-phenotyped cohorts of ARDS, sepsis, trauma, and acute pancreatitis patients. Blood was collected in red-top and EDTA-treated tubes for serum and plasma respectively, centrifuged within 1 h from sample collection (2000 Å ~ g for 20 min, RCF), and stored at − 80 °C. In the first cohort, plasma and serum concentrations of eNAMPT were quantified using an in-house Enzyme-linked Immunosorbent Assay (ELISA) [22]. In the second study cohort, circulating levels of eNAMPT in plasma were quantified using MSD-Uplex assay electrochemiluminescent immunoassay predesigned panel from MesoScale (Meso Scale Discovery, MSD®) [23].

### Statistical analysis

#### Descriptive statistics

Standard descriptive statistics were used to summarize the data for all cohorts. For the continuous variables, the mean, and standard deviation were calculated for the entire data. Counts and percentages were calculated for the categorical variables.

#### Comparison of circulating eNAMPT levels in disease and controls

For patients with sepsis, trauma, and ARDS (one-time measurements), a log base 2 variance stabilizing transformation was performed on the circulating eNAMPT measurements and linear regression models were used to compare the log-transformed eNAMPT measurement (response variable) between each disease category versus controls. Additionally, each log-transformed eNAMPT measurement was fit to the disease severity (for example control vs. sepsis, septic shock; or control vs. trauma in lower, upper, and combined upper and lower parts of the torso).

For the serum pancreatitis samples with repeated eNAMPT measurements, a similar log base 2 variance stabilizing transformation was performed on each eNAMPT measurements (i.e., first and second measurements). The transformed eNAMPT measurement was fit to the disease category (control, pancreatitis) as well as the time of measurement to ascertain if there was a difference in eNAMPT levels between the two groups or if the measurements varied because of the time of measurement. Generalized estimating equations (GEE) models were then used to fit the log-transformed eNAMPT level comparing controls to mild, moderate, and severe acute pancreatitis. Timing of measurement was included in the model to ascertain whether the severity of pancreatitis is affected by eNAMPT levels. We plotted receiver operating curves (ROC) and calculated area under the curves (AUC) to determine how well circulating eNAMPT levels distinguished acute pancreatitis from normal controls. The ROC analysis results were interpreted as follows: AUC < 0.70, low diagnostic accuracy; range of 0.70 – 0.90, moderate diagnostic accuracy; and AUC ≥ 90, high diagnostic accuracy.

#### Determination of optimal disease-distinguishing plasma/serum eNAMPT level

After log base 2 variance stabilizing transformation of all plasma/serum eNAMPT measurements for each cohort and corresponding controls, the mean and standard deviation (SD) of the combined dataset was calculated. We used an empirical rule to calculate cutoffs at 0, 0.25, 0.5, 0.75, -0.25 SDs. Each cutoff was then converted back to the original units. For each cutoff value, a 2X2 contingency table was generated for each cohort and corresponding controls and the sensitivity, specificity, positive predictive value (PPV), and negative predictive values (NPV) were calculated.

Statistical analysis was performed using Stata version 16.1 (StataCorp) and GraphPad Prism version 8.0 (Sn Diego Ca.) software.

## Results

### Characteristics of the cohorts

The demographic and clinical characteristics of each cohort are presented in Table 1. The first cohort (in house ELISA assay) included specimens from 690 subjects and the second cohort (MSD assay) included samples from 276 subjects. The trauma group comprised 67 samples from all male US Army soldiers with field trauma, mean age of 25 years. Twenty-one trauma subjects experienced upper body injury, 31 with lower body injury, and 13 exhibited a combination of upper and lower body injury. The sepsis group from South Korea represented the oldest subset (mean age, 66.5 years). The pancreatitis group consisted of 59 subjects stratified by disease severity (29 mild, 14 moderate, and 16 severe). The highest mortality was observed in the ARDS cohort (21.7%), followed by the South Korean sepsis cohort (19%).

**Table 1:** Baseline characteristics of patient cohorts and control

Disease	N	Age (mean, sd)	Mortality (%)	Sex (male, %)	Race (W/AA/NA/A, %)	Specimen
**First Study Cohort (IH-ELISA assay)**						
Trauma	66	25 ± 5.8	10.4	100.0	NA	plasma
Lower	31	24 ± 4.8	6.5	100	NA	plasma
Upper	20	25 ± 6.7	20	100	NA	plasma
Combined	13	22 ± 8.4	7.6	100	NA	plasma
ARDS	248	50.3 ± 14.3	21.7	51.2	73.7, 18.5, 1.2, 0.4	plasma
Sepsis	123	56.7 ± 16.2	16.2	48.4	66.4, 33.4, 0.75, 0	plasma
Sepsis only	55	54.5 ± 17.7	7.2	54.5	58.8, 40, 1.8, 0	plasma
Septic shock	68	59.5 ± 14.5	23.5	41.2	72, 28, 0, 0	plasma
Sepsis (SK)	100	66.5 ± 13.5	19	62.0	0, 0, 0, 100	serum
Sepsis only	20	60.3 ± 15.2	5	80.0	0, 0, 0, 101	serum
Septic shock	80	66.2 ± 12.7	21.2	58.7	0, 0, 0, 102	serum
Pancreatitis	59	51 ± 18.4	3.3	45.8	88.1, 6.7, 7.1, 0	serum
Mild	29	50.8 ± 20.2	0	37.9	82.7, 10.3, 0, 0	serum
Moderate	14	45.8 ± 16.6	0	42.9	85.7, 7.1, 7.1, 0	serum
Severe	16	56.3 ± 15.2	12.5	62.5	100, 0, 0, 0	serum
Healthy controls	70	62 ± 14	0	70	82.8, 10, 1.4, 0	plasma
Healthy controls	31	57.5 ± 20.9	0	32.3	83.9, 9.7, 3.2, 0	serum
**Second Study Cohort (MSD assay)**						
ARDS	100	55.4 ± 15.3	37	52	88, 3, 7, 1	plasma
Sepsis	98	55.5 ± 17.6	14	48	84, 13, 1, 1	plasma
Healthy controls	78	55.2 ± 16.75	0	57.7	83, 10.2, 1, 0	plasma

Baseline characteristics of the first and second study cohorts included in the analysis. Age is reported in mean ± standard deviation (sd), mortality, gender, and race are shown as percentages. Race *abbreviations*: White (W), African American (AA), Native American (NA), Asian (A) South Korean (SK). Pancreatitis Classification: Mild: no organ failure/ systemic complications. Moderate: transient organ failure local complications or exacerbation of comorbidities. Severe: persistent organ failure (> 48H)

### First cohort (in house eNAMPT ELISA assay):

#### Circulating eNAMPT in acute pancreatitis

**Fig. 2:**
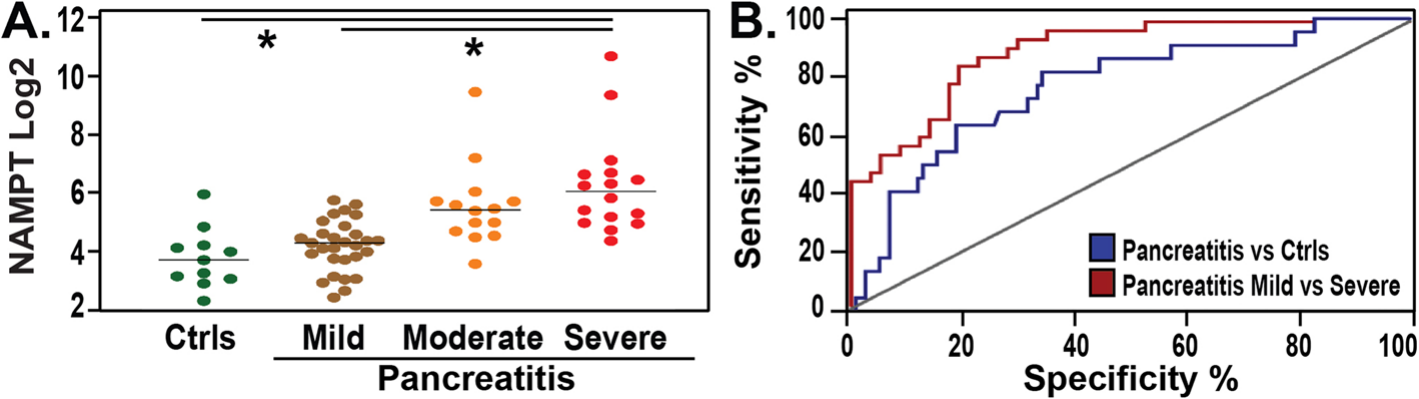
First Study Cohort: Pancreatitis circulating eNAMPT levels are significantly elevated in acute pancreatitis compared to controls. Positive correlation with disease severity. **A** Y-axis represents log base 2 transformation of plasma eNAMPT values; X-axis group comparison: healthy controls and pancreatitis by severity groups – mild, moderate, severe. Comparisons of medians between pancreatitis and healthy controls and pancreatitis by severity subgroups significantly differ (*p*-value < 0.01) Kruskal–Wallis test. eNAMPT levels were significantly different in the three severity categories of acute pancreatitis, with significantly higher levels in severe pancreatitis compared to mild pancreatitis, (median 67.7 vs 17.6 ng/ml, *p* < 0.01).**B** Pancreatitis ROC plots and the corresponding AUCs. eNAMPT distinguishes acute pancreatitis from healthy subjects (blue) (AUC = 0.74, 95% confidence interval: 0.62–0.86, p 0.009). High diagnostic accuracy for eNAMPT levels was observed in patients with severe (red) pancreatitis compared to mild (brown) pancreatitis (AUC = 0.92, 95% confidence interval: 0.85—1.0, *p*-value < 0.01)

When compared to controls, circulating eNAMPT levels were significantly higher in patients with acute pancreatitis (regardless of severity), (median 20.4 ng/ml, vs 26.84 ng/ml, respectively, *p* < 0.01) (Fig. 2A). Furthermore, the circulating eNAMPT levels were significantly different in the three severity categories of acute pancreatitis, with higher levels observed among subjects with severe pancreatitis compared to mild pancreatitis, (median 67.7 vs 17.6 ng/ml, *p* < 0.01) and higher in moderate pancreatitis compared to mild (median 40.4 ng/ml vs 17.6 ng/ml, *p* < 0.01), nonetheless, eNAMPT levels were not significantly different when moderate vs. severe pancreatitis subjects were compared (*p*-value = 0.24). Results of the generalized estimating equations (GEE) models fit the log-transformed eNAMPT level comparing controls to mild, moderate, and severe acute pancreatitis are shown in Table 2. Serum eNAMPT at baseline significantly distinguished patients with acute pancreatitis (regardless of severity) from healthy controls (AUC = 0.74, 95% confidence interval: 0.62–0.86, *p* = 0.009). High diagnostic accuracy for eNAMPT levels was observed in patients with severe pancreatitis compared to mild pancreatitis (AUC = 0.92, 95% confidence interval: 0.85—1.0, *p*-value < 0.01) (Fig. 2B).

**Table 2:** Circulating eNAMPT levels elevated in acute pancreatitis compared to healthy controls

**Predictor**	**Log (eNAMPT ng/ml)**		
	**Estimates**	**95% CI**	***P*-Value**
Intercept	2.68	2.14 – 3.17	< 0.0001
Mild Pancreatitis	0.196	-0.22 – 0.85	0.24
Moderate Pancreatitis	1.23	0.59 – 1.81	< 0.0001
Severe Pancreatitis	1.66	1.21 – 2.54	< 0.0001

Results of the generalized estimating equations (GEE) models fit the log-transformed eNAMPT level comparing controls (reference) to mild, moderate, and severe acute pancreatitis. Patients with moderate and severe acute pancreatitis exhibited significantly elevated eNAMPT levels when compared to controls (*P*-value < 0.0001), however, eNAMPT levels were not significantly different between subjects with mild pancreatitis and controls (*P*-value = 0.24)

#### Circulating eNAMPT levels in sepsis and septic shock

**Fig. 3:**
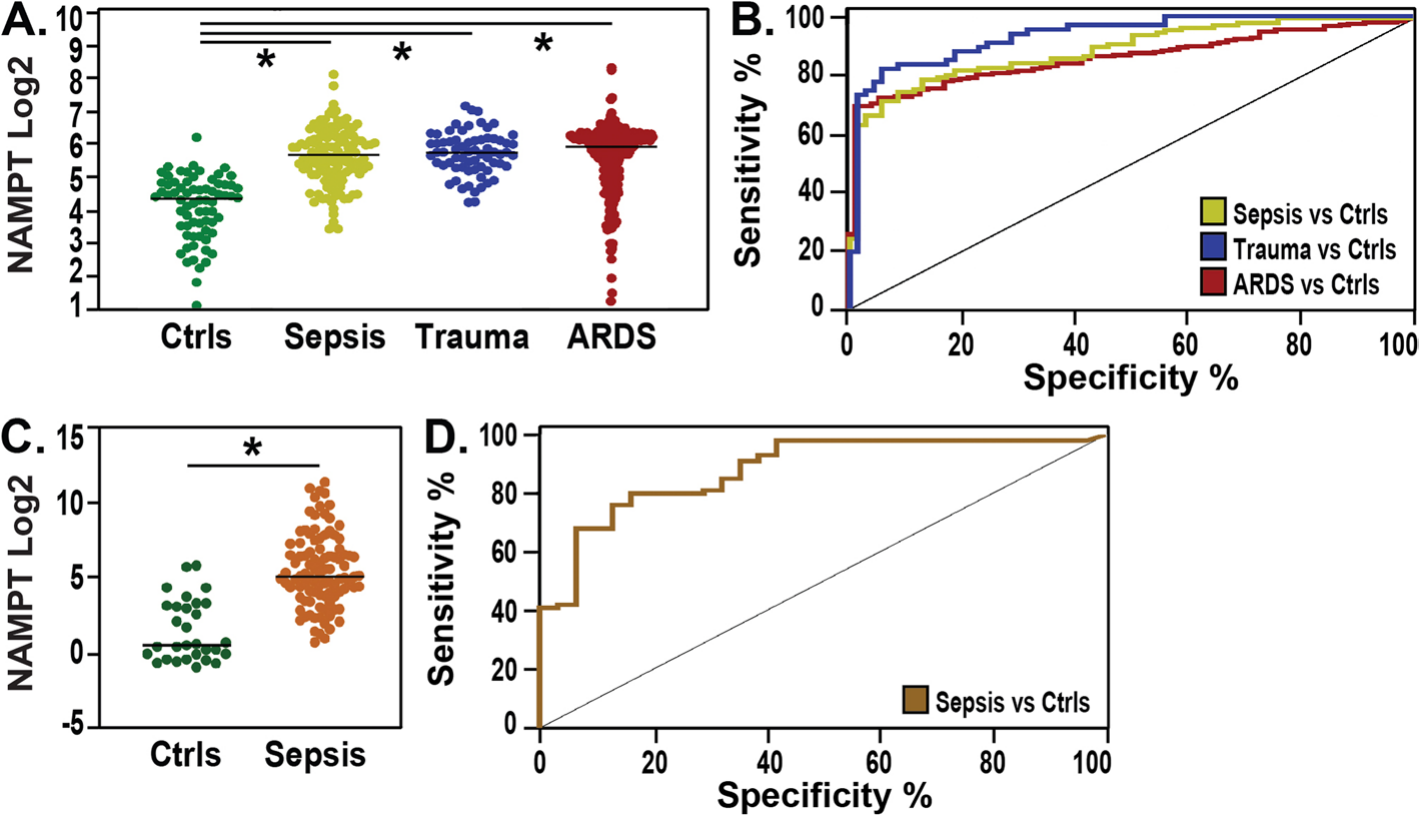
First Study Cohort: Circulating eNAMPT are elevated in ARDS, Sepsis, and Trauma subjects compared to healthy controls. Data represented in Panels **A** and **B** reflect eNAMPT measurements in plasma samples and Panels **C** and **D** reflect eNAMPT measurements in serum samples. In Panels **A** and **C**, the Y-axis represents eNAMPT Log 2 transformed values; X-axis group comparison: healthy controls (green), ARDS (red), Sepsis (yellow or brown), and Trauma (blue). **B** ROC plot and the corresponding AUC show that eNAMPT (at baseline) distinguishes: i) ARDS (red) from healthy controls (AUC = 0.86, 95% confidence interval: 0.82–0.90, *p*-value < 0.001); ii) sepsis (yellow) from healthy controls (AUC = 0.89, 95% confidence interval: 0.85–0.93 *p* < 0.001) and iii) trauma (blue) vs healthy controls (AUC = 0.94, 95% confidence interval: 0.90–0.97 *p* < 0.001. **D** ROC plot and AUC of eNAMPT in serum of septic subjects, AUC 0.88, 95% CI 0.81–0.95, *p* < 0.01

There were two independent groups of sepsis/septic shock and controls reflecting the two ELISA assays utilized. In the first group, median plasma eNAMPT levels were significantly higher in 123 subjects with sepsis or septic shock when compared to the 70 healthy controls (51.5 ng/ml vs 20.4 ng/ml, *p* < 0.01) (Fig.3A). There was no significant difference in plasma eNAMPT levels between patients with sepsis and those with septic shock (median 47.2 ng/ml vs 51.5 ng/ml; *p* = 0.4). Similarly, there was no significant difference in circulating plasma eNAMPT levels between sepsis survivors and non-survivors (*p*-value = 0.35). Plasma eNAMPT significantly distinguished patients with sepsis and septic shock from healthy controls (AUC = 0.89, 95% confidence interval: 0.85—0.93; *p* < 0.01) (Fig. 3B). In the second group (South Korean (SK) subjects), median serum eNAMPT levels were significantly higher in the 100 subjects with sepsis or septic shock compared to the 31 healthy controls (34.7 ng/ml vs. 1.3 ng/ml, *p* < 0.01) (Fig. 3C). The plasma eNAMPT levels in Group 1 accurately distinguished patients with sepsis/septic shock from healthy controls, with a median of 34.68 ng/ml in sepsis vs 1.27 ng/ml in controls (AUC 0.93, 95% CI 0.86–0.99, *p* < 0.01) (Fig. 3C). Similarly, serum eNAMPT levels in the SK Group 2, were not significantly different when comparing SK subjects with sepsis (*n* = 20) to septic shock (*n* = 80) (*p*-value = 0.15) or comparing sepsis/septic shock survivors to non-survivors (*p*-value = 0.5). Serum eNAMPT levels accurately and robustly distinguished sepsis subjects from controls (AUC 0.88, 95% CI 0.81–0.95, *p* < 0.01) (Fig.3D).

#### Circulating eNAMPT in trauma

When compared to controls, median plasma eNAMPT levels were significantly higher in trauma subjects (20.4 ng/ml vs 54 ng/ml; *p* < 0.01) (Fig. 3a). There was no significant correlation between body parts affected by trauma (upper, lower, or combined) and circulating eNAMPT levels. Plasma eNAMPT at baseline significantly distinguished patients with acute trauma from healthy controls with high diagnosis accuracy (AUC = 0.94, 95% CI: 0.90—0.97; *p *< 0.01) (Fig. 3B).

#### Circulating eNAMPT in ARDS

When compared to controls, median plasma eNAMPT levels were significantly higher in patients with ARDS (20.4 ng/ml vs. 60.7 ng/ml; *p* < 0.01 respectively). Plasma eNAMPT at baseline significantly distinguished patients with ARDS from healthy controls (AUC = 0.86, 95% confidence interval: 0.82—0.90, *p* < 0.01) (Fig. 3B).

### Second Study Cohort (MSD assay)

#### Circulating eNAMPT in sepsis and ARDS patients

**Fig. 4:**
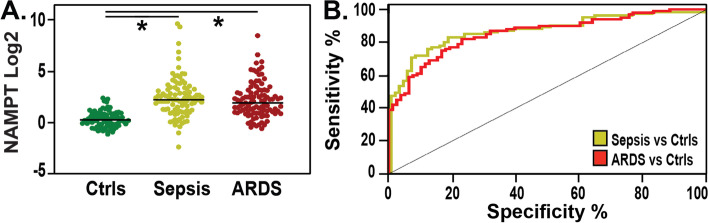
Second Study Cohort: Circulating eNAMPT levels are significantly elevated in Sepsis and ARDS subjects compared to healthy controls. In Panel **A** Y-axis represents eNAMPT Log 2 transformed values; X-axis group comparison: healthy controls (green), ARDS (red), Sepsis (yellow). Median plasma eNAMPT values were significantly higher in ARDS and Sepsis compared to healthy controls—3.78 ng/ml, 4.7 ng/ml, and 1.2 ng/ml respectively; *p* < 0.01. **B** ROC Curve. Plasma eNAMPT levels accurately distinguished healthy controls from ARDS (red), AUC 0.85 (95% CI 0.8–0.9, *p*-value < 0.0001), and healthy controls from Sepsis (yellow), AUC 0.87 (95% IC 0.82–0.92 *p*-value < 0.0001). No significant differences between ARDS and Sepsis

In this cohort that utilized the MSD assay, median plasma eNAMPT levels were significantly higher in sepsis patients when compared to controls (4.7 ng/ml vs. 1.2 ng/ml; *p* < 0.01) and in ARDS patients when compared to controls (3.78 ng/ml vs. 1.2 ng/ml; *p* < 0.01) (Fig. 4A). Plasma eNAMPT at baseline significantly distinguished patients with ARDS and healthy controls (AUC = 0.85, 95% CI: 0.8–0.9, *p*-value < 0.01), and patients with sepsis and healthy controls (AUC 0.87 95% CI: 0.82–0.92, *p*-value < 0.01) (Fig. 4B). No significant differences were present comparing ARDS and Sepsis subjects.

#### Circulating eNAMPT cut-off determinations in critically ill subjects and controls

For the pancreatitis patients utilizing the IH-ELISA assay, circulating eNAMPT cutoff values of 26 ng/ml and 32.5 ng/ml exhibited the best estimates of sensitivity, specificity, NPV, and PPV (Supplemental Table 1). For the sepsis patients, eNAMPT cutoffs of 33.8 ng/ml, and 41.4 ng/ml exhibited the best estimates of sensitivity, specificity, NPV, and PPV (Supplemental Table 2). For the trauma patients, eNAMPT cutoffs of 36.6 ng/ml and 44.4 ng/ml demonstrated the best estimates of sensitivity, specificity, NPV, and PPV (Supplemental Table 3). For the ARDS patients, the eNAMPT cutoffs 38.1 ng/ml and 46.9 ng/ml, exhibited the best estimates of sensitivity, specificity, NPV, and PPV (Supplemental Table 4). Taken together, a plasma eNAMPT level of 35—45 ng/ml (IH-ELISA assay) would be a reasonable cutoff point to distinguish controls from patients with acute inflammatory conditions such as sepsis, trauma, acute pancreatitis, and ARDS.

## Discussion

The activation of evolutionarily-conserved inflammatory cascades, such as the pathogen-receptor recognition TLR4 pathway, triggered by multiple acute inflammatory conditions such as sepsis (viral, bacterial, fungal), acute pancreatitis, and trauma directly contribute to the multi-organ failure and high mortality associated with ARDS [24, 25]. In addition to the intense inflammatory cascade that characterizes ARDS pathogenesis, endothelial and epithelial cell injury, dysregulated coagulation, apoptosis, and fibrosis are prominent features [26]. Accordingly, numerous potential biomarkers are generated by the acute dysregulation of multiple biochemical and cellular pathways characteristic of ARDS pathobiology [26, 27]. Extracellular NAMPT was identified as a damage-associated molecular pattern protein (DAMP) [11] and potential biomarker and druggable target based on extensive research including preclinical mechanistic, genomic, and multi-species ARDS models [4, 11, 28, 29]. The transcriptional regulation and blood/lung protein expression of NAMPT is highly induced by multiple ARDS-relevant stimuli including bacterial infection, shock, trauma, hypoxia, and excessive mechanical stress [30–33]. Upon binding to TLR4, eNAMPT elicits a profound cytokine release that is mediated by proinflammatory transcription factors such as NFκB and leads to increased vascular permeability and ultimately multi-organ dysfunction [11, 30]. The role of eNAMPT as a potential stand alone diagnostic or prognostic biomarker in ARDS has not previously been demonstrated although eNAMPT was among a panel of six biomarkers that predicted mortality in ARDS [12]. An ideal diagnostic biomarker for ARDS would identify early stages of the syndrome, minimize disease heterogeneity, reflect the natural history of the syndrome, and be a potential target for a clinical trial [34]. A prognostic biomarker would provide information that addresses the overall outcome of ARDS and be potentially useful in stratifying patients for enrollment in clinical trials, thus enhancing the ability to detect beneficial effects from novel therapies [34].

We have confirmed and validated, for the first time, that circulating eNAMPT is a potential biomarker in ARDS and several ARDS-predisposing systemic acute inflammatory conditions including acute pancreatitis, sepsis, septic shock, and trauma. In addition to demonstrating that median circulating eNAMPT levels were significantly higher in ARDS and these ARDS-inducing acute inflammatory conditions when compared to healthy controls, we identified the range of eNAMPT values between 26 ng/ml and 33 ng/ml based on our in-house colorimetric ELISA assay to represent the best possible cut-off for distinguishing patients from healthy controls.

The translational utility of circulating eNAMPT as a biomarker in ARDS include early diagnosis, minimizing disease heterogeneity, and facilitating the selection of subjects for enrollment in clinical trials, especially for therapies targeting this pathway [29, 35]. An interesting finding in our analysis was the positive correlation between circulating eNAMPT levels and severity of acute pancreatitis. This also highlights the potential prognostic utility of eNAMPT as a biomarker. We did not replicate this positive correlation with disease severity in the sepsis, trauma, and ARDS cohorts, likely because of suboptimal classification of disease severity, non-standardized timing of specimen collection, and heterogeneity of cohorts.

The strengths of our report include, the inclusion of a large and diverse population of patients and controls in the first cohort in plasma and serum, and use of two novel complementary assays, the MSD-Uplex assay electrochemiluminescent immunoassay, MesoScale (Meso Scale Discovery, MSD®) [23] and our validated in-house ELISA assay [31]. Our findings are also strengthened by the consistency across all the cohorts where circulating eNAMPT levels were significantly higher in patients with acute systemic inflammatory states (sepsis, trauma, acute pancreatitis, and ARDS) when compared to controls. The clear discriminatory power of eNAMPT in acute severe pancreatitis is not only innovative and clinically relevant since ARDS is very common in patients with severe acute pancreatitis. Our results identified eNAMPT as a novel reliable marker of pancreatitis severity. Early interventions that allow differentiating between mild and severe pancreatitis are clinically relevant for the prognosis and to avoid athe potential development of ARDS and multiple organ dysfunction.

The limitations of our analysis, are inherent with the use of previously bio-banked specimens, one of them is the heterogeneity of our cohorts as shown by variability in mortality rates and available phenotypic variables. We did not compare the diagnostic performance of circulating eNAMPT to other diagnostic biomarkers in ARDS, such as soluble receptor for advanced glycation end products (sRAGE) [36] and Angiopoeitin-2 [37, 38]. We had limited information on generic severity of illness scales such as APACHE IV scores, sequential organ assessment (SOFA) scores or multiple organs dysfunction (MOD) scores, which limited the ability to better classify the patients within each cohort and further refine the diagnostic value of eNAMPT.

## Conclusion

In summary, we have shown that circulating eNAMPT is significantly elevated in ARDS and ARDS-predisposing conditions and represents a promising biomarker with potential utility as a stratification tool for enrollment of subjects in clinical trials targeting eNAMPT neutralization.

## Supplementary Information

**Additional file 1**.

**Additional file 2**.

**Additional file 3**.

**Additional file 4**.

## Data Availability

The datasets generated and analyzed during the current study are available within the article supplementary information files.

## Abbreviations

ARDS: Acute Respiratory Distress Syndrome
NAMPT: Nicotinamide phosphoribosyl transferase
W: White
AA: African American
NA: Native American
SK: South Korean
PPV: Positive predictive value
NPV: Negative predictive values
ROC: Receiver operating curves
AUC: Calculated area under the curves
