# Supplementary material for: Circulating eNAMPT as a biomarker in the critically ill: acute pancreatitis, sepsis, trauma, and acute respiratory distress syndrome
Source: BMC Anesthesiol. 2022 Jun 15;22:182. doi: 10.1186/s12871-022-01718-1 (PMC9198204; doi:10.1186/s12871-022-01718-1)
Supplement: Supplementary file 1 — Additional file 1. [file 12871_2022_1718_MOESM1_ESM.docx]

| **Acute Pancreatitis Cohort** | | | | |
| --- | --- | --- | --- | --- |
| eNAMPT value | Statistical Test | Estimate | Lower limit | Upper limit |
| 26 ng/ml | Sensitivity | **0.91** | 0.59 | 0.99 |
|  | Specificity | **0.53** | 0.39 | 0.66 |
|  | NPV | **0.97** | 0.82 | 0.99 |
|  | PPV | **0.27** | 0.21 | 0.34 |
| 32.5 ng/ml | Sensitivity | **0.91** | 0.59 | 0.99 |
|  | Specificity | **0.39** | 0.26 | 0.52 |
|  | NPV | **0.96** | 0.77 | 0.99 |
|  | PPV | **0.22** | 0.18 | 0.27 |
| 40.7 ng/ml | Sensitivity | 0.91 | 0.59 | 0.99 |
|  | Specificity | 0.28 | 0.17 | 0.41 |
|  | NPV | 0.94 | 0.70 | 0.99 |
|  | PPV | 0.20 | 0.16 | 0.24 |
| 51 ng/ml | Sensitivity | 0.91 | 0.59 | 0.99 |
|  | Specificity | 0.26 | 0.16 | 0.40 |
|  | NPV | 0.94 | 0.69 | 0.99 |
|  | PPV | 0.19 | 0.16 | 0.23 |
| 20.7 ng/ml | Sensitivity | 0.72 | 0.39 | 0.94 |
|  | Specificity | 0.68 | 0.55 | 0.80 |
|  | NPV | 0.93 | 0.83 | 0.97 |
|  | PPV | 0.31 | 0.21 | 0.43 |

The pancreatitis study group was assayed with IH-ELISA. Circulating eNAMPT cutoff values of 26 ng/ml and 32.5 ng/ml exhibited the best estimates of sensitivity, specificity, NPV, and PPV.
