# Supplementary material for: Circulating eNAMPT as a biomarker in the critically ill: acute pancreatitis, sepsis, trauma, and acute respiratory distress syndrome
Source: BMC Anesthesiol. 2022 Jun 15;22:182. doi: 10.1186/s12871-022-01718-1 (PMC9198204; doi:10.1186/s12871-022-01718-1)
Supplement: Supplementary file 2 — Additional file 2. [file 12871_2022_1718_MOESM2_ESM.docx]

| **Sepsis Cohort** | | | | |
| --- | --- | --- | --- | --- |
| eNAMPT value | Statistical Test | Estimate | Lower limit | Upper limit |
| 33.8 ng/ml | Sensitivity | **0.87** | 0.77 | 0.94 |
|  | Specificity | **0.79** | 0.71 | 0.86 |
|  | NPV | **0.92** | 0.85 | 0.95 |
|  | PPV | **0.70** | 0.62 | 0.77 |
| 41.4 ng/ml | Sensitivity | **0.99** | 0.92 | 1.0 |
|  | Specificity | **0.63** | 0.54 | 0.72 |
|  | NPV | **0.99** | 0.92 | 1.0 |
|  | PPV | **0.61** | 0.55 | 0.66 |
| 50.6 ng/ml | Sensitivity | 0.99 | 0.92 | 1.0 |
|  | Specificity | 0.50 | 0.41 | 0.60 |
|  | NPV | 0.98 | 0.90 | 1.0 |
|  | PPV | 0.53 | 0.49 | 0.58 |
| 62 ng/ml | Sensitivity | 0.99 | 0.92 | 1.0 |
|  | Specificity | 0.38 | 0.30 | 0.47 |
|  | NPV | 0.98 | 0.87 | 1.0 |
|  | PPV | 0.48 | 0.44 | 0.51 |
| 27.6 ng/ml | Sensitivity | 0.76 | 0.64 | 0.85 |
|  | Specificity | 0.83 | 0.75 | 0.89 |
|  | NPV | 0.86 | 0.80 | 0.90 |
|  | PPV | 0.72 | 0.63 | 0.79 |

In the sepsis cohort eNAMPT cutoffs of 33.8 ng/ml, and 41.4 ng/ml exhibited the best estimates of sensitivity, specificity, NPV, and PPV
