# Supplementary material for: Circulating eNAMPT as a biomarker in the critically ill: acute pancreatitis, sepsis, trauma, and acute respiratory distress syndrome
Source: BMC Anesthesiol. 2022 Jun 15;22:182. doi: 10.1186/s12871-022-01718-1 (PMC9198204; doi:10.1186/s12871-022-01718-1)
Supplement: Supplementary file 3 — Additional file 3. [file 12871_2022_1718_MOESM3_ESM.docx]

| **Trauma Cohort** | | | | |
| --- | --- | --- | --- | --- |
| eNAMPT value | Statistical Test | Estimate | Lower limit | Upper limit |
| 29.7 ng/ml | Sensitivity | 0.81 | 0.70 | 0.90 |
|  | Specificity | 0.88 | 0.78 | 0.95 |
|  | NPV | 0.82 | 0.73 | 0.88 |
|  | PPV | 0.88 | 0.79 | 0.93 |
| 36.3 ng/ml | Sensitivity | **0.91** | 0.82 | 0.97 |
|  | Specificity | **0.84** | 0.73 | 0.92 |
|  | NPV | **0.90** | 0.81 | 0.95 |
|  | PPV | **0.85** | 0.77 | 0.91 |
| 44.4 ng/ml | Sensitivity | **0.98** | 0.92 | 1.0 |
|  | Specificity | **0.66** | 0.53 | 0.77 |
|  | NPV | **0.98** | 0.86 | 1.0 |
|  | PPV | **0.75** | 0.68 | 0.81 |
| 54.3 ng/ml | Sensitivity | 0.99 | 0.92 | 1.0 |
|  | Specificity | 0.48 | 0.35 | 0.60 |
|  | NPV | 0.97 | 0.82 | 1.0 |
|  | PPV | 0.66 | 0.61 | 0.71 |
| 24.3 ng/ml | Sensitivity | 0.64 | 0.52 | 0.75 |
|  | Specificity | 0.96 | 0.87 | 0.99 |
|  | NPV | 0.72 | 0.65 | 0.78 |
|  | PPV | 0.94 | 0.83 | 0.98 |

In the trauma cohort, eNAMPT cutoffs of 36.6 ng/ml and 44.4ng/ml demonstrated the best estimates of sensitivity, specificity, NPV, and PPV
