# Supplementary material for: Circulating eNAMPT as a biomarker in the critically ill: acute pancreatitis, sepsis, trauma, and acute respiratory distress syndrome
Source: BMC Anesthesiol. 2022 Jun 15;22:182. doi: 10.1186/s12871-022-01718-1 (PMC9198204; doi:10.1186/s12871-022-01718-1)
Supplement: Supplementary file 4 — Additional file 4. [file 12871_2022_1718_MOESM4_ESM.docx]

| **ARDS Cohort** | | | | |
| --- | --- | --- | --- | --- |
| eNAMPT value | Statistical Test | Estimate | Lower limit | Upper limit |
| 38.1 ng/ml | Sensitivity | **0.94** | 0.86 | 0.98 |
|  | Specificity | **0.72** | 0.66 | 0.78 |
|  | NPV | **0.98** | 0.94 | 0.99 |
|  | PPV | **0.49** | 0.44 | 0.54 |
| 46.9 ng/ml | Sensitivity | **0.99** | 0.92 | 1.0 |
|  | Specificity | **0.66** | 0.60 | 0.72 |
|  | NPV | **0.99** | 0.96 | 1.0 |
|  | PPV | **0.45** | 0.40 | 0.49 |
| 57.6 ng/ml | Sensitivity | 0.99 | 0.92 | 1.0 |
|  | Specificity | 0.53 | 0.47 | 0.60 |
|  | NPV | 0.99 | 0.95 | 1.0 |
|  | PPV | 0.38 | 0.34 | 0.41 |
| 70.9 ng/ml | Sensitivity | 0.98 | 0.92 | 1.0 |
|  | Specificity | 0.35 | 0.29 | 0.41 |
|  | NPV | 0.99 | 0.92 | 1.0 |
|  | PPV | 0.30 | 0.28 | 0.32 |
| 31 ng/ml | Sensitivity | 0.83 | 0.72 | 0.91 |
|  | Specificity | 0.78 | 0.73 | 0.83 |
|  | NPV | 0.94 | 0.91 | 0.96 |
|  | PPV | 0.52 | 0.45 | 0.58 |

In ARDS patients, the eNAMPT cutoffs 38.1 ng/ml and 46.9 ng/ml, exhibited the best estimates of sensitivity, specificity, NPV, and PPV
